# Supplementary material for: Effervescence in a binary mixture with nonlinear non-reciprocal interactions
Source: Nat Commun. 2025 Aug 7;16:7310. doi: 10.1038/s41467-025-61728-8 (PMC12332134; doi:10.1038/s41467-025-61728-8)
Supplement: Supplementary file 1 — Supplementary Information [file 41467_2025_61728_MOESM1_ESM.pdf]

Supplement: Effervescence in a binary mixture with nonlinear non-reciprocal interactions

Suropriya Saha[1, *] and Ramin Golestanian[1, 2, †]

[1] Max Planck Institute for Dynamics and Self-Organization (MPIDS), D-37077 Göttingen, Germany
[2] Rudolf Peierls Centre for Theoretical Physics, University of Oxford, Oxford OX1 3PU, United Kingdom

(Dated: July 21, 2025)

CONTENTS

I. Analysis of droplets shapes in two dimensions 1
II. Reciprocal granules and droplets in one dimension 1
III. Constructing the phase diagrams 1
IV. Nonlinear NRCH with non zero ϕ_0. 2
V. Dynamics and phase diagram for nonlinear NRCH 3
VI. Stable waves and droplets 4
References 4

I. ANALYSIS OF DROPLETS SHAPES IN TWO DIMENSIONS

The contours of the droplets analysed in Fig. 2b-c in the main text fluctuate rapidly and continuously until they are dissolved. We have analysed the eigenvalues of the moment of inertia tensor associated with the set of points associated with the contour to obtain a sense of the shapes that are found most commonly. Measuring the displacement of the $i-$th point constituting the contour in the two dimensional plane spanned by the unit vectors $\hat{\boldsymbol{x}}$ and $\hat{\boldsymbol{y}}$ and given by $\delta x_i \hat{\boldsymbol{x}} + \delta y_i \hat{\boldsymbol{y}}$ from the centre of the droplet, we define the moment of inertia $\mathbb{I}$ tensor as

$$\mathbb{I} = \sum_i \begin{bmatrix} \Delta y_i \Delta y_i & \Delta x_i \Delta y_i \\ \Delta x_i \Delta y_i & \Delta x_i \Delta x_i \end{bmatrix} \quad \text{(S1)}$$

We define the ratio ℓ that is a ratio $\lambda_{1,2}$ of the matrix in Eq. (S1)

$$\ell = \frac{\max(|\lambda_1|, |\lambda_2|)}{\min(|\lambda_1|, |\lambda_2|)}. \quad \text{(S2)}$$

The distribution of ℓ peaks at a value just a little over 1, around 1.25 meaning that most of the droplets are stabilised. Notice however that there are long tails where the droplets have elongated shapes when they are formed by fusion of reciprocal granules or other droplets.

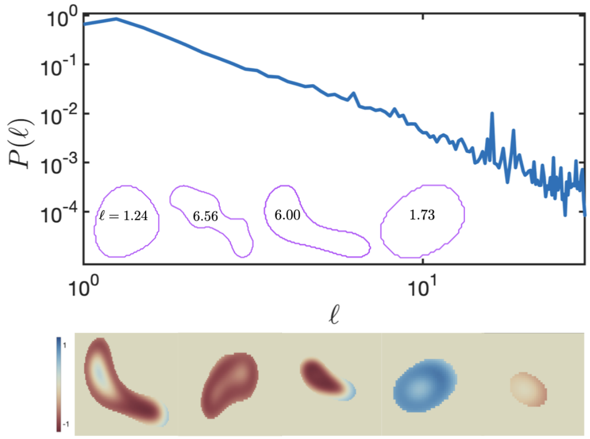

FIG. S1. **Aspect ratio of a droplet.** The distribution shows that the most of the droplets are spherical with an aspect ratio very close to unity. There are however heavy tails as clear from the finite values around $\ell = 10$. We show some of the droplets and the calculated value of ℓ.

II. RECIPROCAL GRANULES AND DROPLETS IN ONE DIMENSION

To correlate the total lifespan of a droplet to its average size we run simulations in one spatial dimension as a similar analysis in two dimensions in challenging. Choosing the parameters $\alpha_0 = \alpha_1 = 5$, we find that the steady-state dynamics consists of a wave that travels with angular frequency $1/5000\ \Gamma^{-1} K^{-1}$. The analysis reveals a simple correlation between droplet size and lifespan (see Figs. S2b-c). Droplets are nucleated and annihilated at time scales that are small compared to the timescale of $5000\ \Gamma K$ associated with the travelling waves as seen in Fig. S2.

III. CONSTRUCTING THE PHASE DIAGRAMS

The phase diagram in two dimensions is calculated by running simulations for $\phi_0 = 0$ at 61^2 discrete points in the $(\alpha_1 - \alpha_0)$ plane. The simulations are performed on a grid of size 201×201. The quantities used to determine the phase boundaries are as follows. The cumulative density $C_{0.1} = \int_0^{0.1} \mathrm{d}|\phi| P(|\phi|)$ is used to determine the boundary between ravelling waves and the states with effervescence, see Fig. S4a. The peak height

* suropriya.saha@ds.mpg.de
† ramin.golestanian@ds.mpg.de

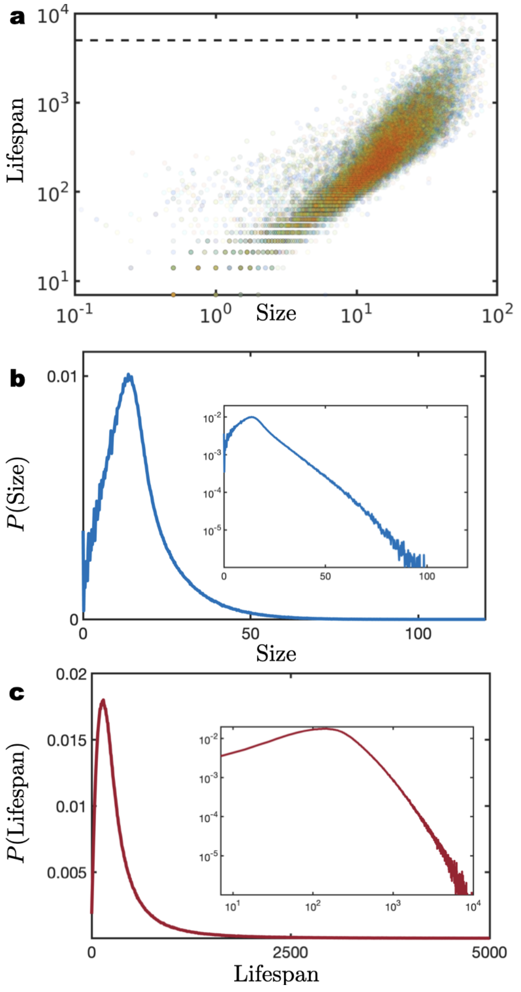

FIG. S2. **Statistics for the lifetime of a droplet versus its size.** **a**, The time for which a droplet persists once it is nucleated is plotted against its average size during its lifetime. The calculations are done in one dimension. The data shows that on an average, a droplet lifetime correlates linearly with droplet size, a larger droplet persists for longer presumably because it can grow due to Oswald ripening at the expense of other droplets. The markers of different colours correspond to simulations carried out with different initial conditions. **b**, There are large fluctuations in the lifetime and the sizes. These fluctuations are quantified by collecting data for more than 3 million droplets. The results for size shows oscillations at small size that are not as well defines as those reported in two dimensions and presented in the main text. This could be related to the differences in dynamics in one and two dimensions. The inset shows a size is selected. **c**, Droplet lifetime distribution shows a finite peak and mirrors the oscillations in size as well. The distribution has a heavy tail as apparent from the inset.

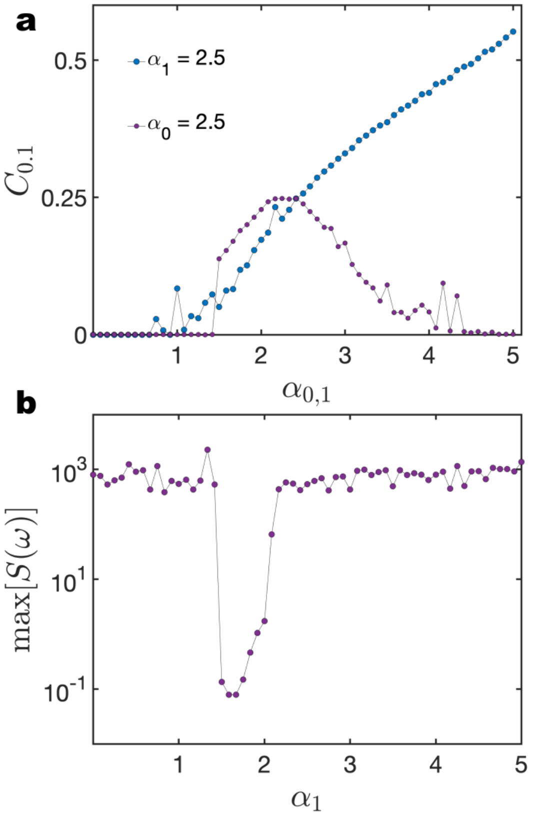

FIG. S3. **Measures used to construct the state diagram.** **a**, The cumulative probability density is plotted by varying $\alpha_{0,1}$ for $\alpha_{1,0}$ fixed at 2.5. $C_{0.1}$ is effective in determining the boundary between travelling waves and the effervescent state as evidence by the discontinuous change seen in the curve for $\alpha_0 = 2.5$. **b**, The maximum of $S(\omega)$ is at least two orders of magnitude higher in the steady-states with a wave compared to the effervescent state - a quantity used to determine the boundary between effervescence and effervescent waves.

of the power spectrum is measures as the height above the average: The power spectrum $S(\nu)$ is calculated as a function of frequency. The maximum height of $S(\omega)$ is used as the quantity to distinguish between effervescence and effervescent waves.The peak height dips several orders of magnitude on crossing the boundary from travelling waves to effervescence or from effervescent waves to travelling waves; making it a good order parameter for determining the phase boundaries, see Fig. S4b.

IV. NONLINEAR NRCH WITH NON ZERO ϕ_0.

At non-vanishing average composition the equation of motion for the $U(1)$ invariant model introduced in the main text loses its simplicity. It can be re-written by substituting $\phi \equiv \phi + \phi_0$ in Eq. (5) to obtain the dynamics for the redefined ϕ subject to the constraint $\langle \phi \rangle = 0$.

$$\partial_t \phi = \nabla^2 \left[-(1 - i\alpha_0 + 2(1 - i\alpha_1)|\phi_0|^2)\phi + (1 - i\alpha_1)\phi_0^2\phi^* + (1 - i\alpha_1)|\phi|^2(\phi + 2\phi_0) + (1 - i\alpha_1)\phi_0^*\phi^2 \right] - K\nabla^4\phi. \quad \text{(S3)}$$

Notice that Eq. (S3) has terms that break the gauge symmetry. Eq. (S3) can be cast in the form similar to Eqs. (1-2) in the main text redefining the bulk free energy f and

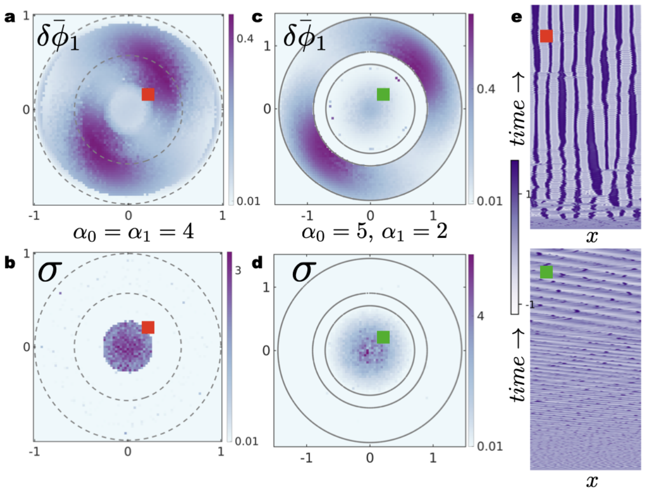

FIG. S4. **Steady-states in one dimension in the composition plane.** **a**, Deviation from a homogeneous mixture quantified by $\delta\bar{\phi}_1$ is plotted for the same values of $\alpha_{0,1}$ as chosen to illustrate dynamics in two dimensions, see Fig. 6 in the main text. $\delta\bar{\phi}_1$ changes rapidly from a smaller value near the centre of the circle to a larger value on moving radially away indicating a change from dynamics dominated by phase separation. **b** σ follows trends opposite $\delta\bar{\phi}_1$ corresponding to travelling waves and oscillations close to $\phi_0 = 0+i0$ and phase separated domains with pulsating interfaces on moving away. **c-d** Shows similar plots for a different set of $\alpha_{0,1}$ indicated in the figure. **e-f** show examples of kymographs for ϕ_0 at the points indicated.

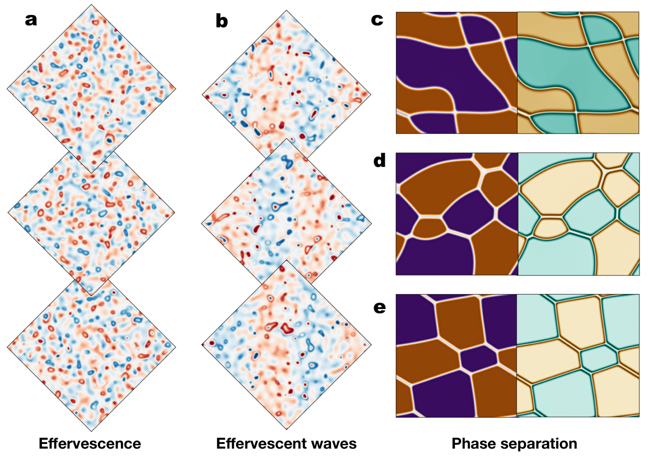

FIG. S5. **Dynamical steady-states of the nonlinear non-reciprocal Cahn-Hilliard.** **a**, Snapshots of the density field ϕ_1 at times $t = 0, 100, 200$ for $\alpha_0 = \alpha_1 = 3$. **b**, Same as Fig. S5a for $\alpha_0 = \alpha_1 = 4$. **c**, Phase separation for $\alpha_0 = \alpha_1 = 1.73$, and $\chi = -0.276$. **d**, Phase separation for $\alpha_0 = \alpha_1 = 3.57$ and $\chi = -0.7$. **e**, Phase separation for $\alpha_0\alpha_1 = 4$ and $\chi = 1.9$. The composition is fixed at $\langle\phi\rangle = 0 + i0$.

introducing general coefficients α_{12} and α_{12}

$$\partial_t\phi_1 = \nabla^2\left[\frac{\partial f}{\partial\phi_1} + \alpha_{12}(\phi_1,\phi_2)\phi_2\right] - K_1\nabla^4\phi_1,$$
$$\partial_t\phi_2 = \nabla^2\left[\frac{\partial f}{\partial\phi_2} + \alpha_{21}(\phi_1,\phi_2)\phi_1\right] - K_2\nabla^4\phi_2, \quad \text{(S4)}$$

where f is

$$\begin{aligned} f =& -\left[1 - 2|\phi_0|^2 + \mathrm{Re}(\phi_0^2) + \alpha_1\mathrm{Im}(\phi_0^2)\right]\frac{\phi_1^2}{2} \\ &+ \left[3\mathrm{Re}(\phi_0) + \alpha_1\mathrm{Im}(\phi_0)\right]\frac{\phi_1^3}{3} \\ =& \frac{(\phi_1^2+\phi_2^2)^2}{4} + \mathrm{Re}(\phi_0)\phi_1\phi_2^2 + \mathrm{Im}(\phi_0)\phi_1\phi_2^2 - \mathrm{Im}(\phi_0^2)\phi_1\phi_2 \\ &- \left[1 - 2|\phi_0|^2 + \mathrm{Im}(\phi_0^2) - \alpha_1\mathrm{Re}(\phi_0^2)\right]\frac{\phi_2^2}{2} \\ &+ \left[3\mathrm{Im}(\phi_0) + \alpha_1\mathrm{Re}(\phi_0)\right]\frac{\phi_2^3}{3}, \end{aligned} \quad \text{(S5)}$$

where the first two lines and the last two lines is the effective self interaction terms for scalar fields 1 and 2 respectively while the third term shows the effective interactions. Notice that the passive phase separation dynamics is no longer identical for the two fields. Also we have a linear coefficient of reciprocity given by the term quadratic in the fields. We point out these details to stress again on the point that the study of the effect of composition naturally leads to the exploration of a more general model with visibly and quantitatively similar results as for vanishing ϕ_0 showing that effervescence is a general and robust phenomenon.

Building on the same theme, the coefficients of non-reciprocal interactions are as follows

$$\begin{aligned} \alpha_{12}(\phi_1,\phi_2) =& \left[-\alpha_0 + 2\alpha_1|\phi_0|^2 - \alpha_1\mathrm{Re}(\phi_0^2)\right] \\ &+ 2\alpha_1\mathrm{Re}(\phi_0)\phi_1 + 3\alpha_1\mathrm{Im}(\phi_0)\phi_2 + \alpha_1(\phi_1^2+\phi_2^2) \\ \alpha_{21}(\phi_1,\phi_2) =& \left[\alpha_0 - 2\alpha_1|\phi_0|^2 - \alpha_1\mathrm{Im}(\phi_0^2)\right] \\ &- 2\alpha_1\mathrm{Re}(\phi_0)\phi_2 - 3\alpha_1\mathrm{Re}(\phi_0)\phi_1 - \alpha_1(\phi_1^2+\phi_2^2) \end{aligned} \quad \text{(S6)}$$

We note the form of non-reciprocal coefficient is more general than before. In fast all terms that can be allowed by symmetry are generated re-enforcing the statements in the previous paragraph about the generality and thus simplicity of our model.

We now turn to the verification of the results of linear stability in one dimension. The results of the stability analysis are verified numerically by running 81×81 simulations keeping $\alpha_{0,1}$ fixed and by varying $\phi_{1,2}$ (see Fig. 8 in the main text). For $\alpha_0 = 5$ and $\alpha_1 = 2$, $\phi_{1,2}$ are varied in the range -1 to 1 while for $\alpha_0 = 4$ and $\alpha_1 = 4$ they are varied in the range -1.5 to 1.5.

V. DYNAMICS AND PHASE DIAGRAM FOR NONLINEAR NRCH

We observe effervescence for f

$$f = -\frac{\phi_1^2}{2} + \frac{\phi_1^4}{4} - \frac{\phi_2^2}{2} + \frac{\phi_2^4}{4} + \chi\phi_1\phi_2. \quad \text{(S7)}$$

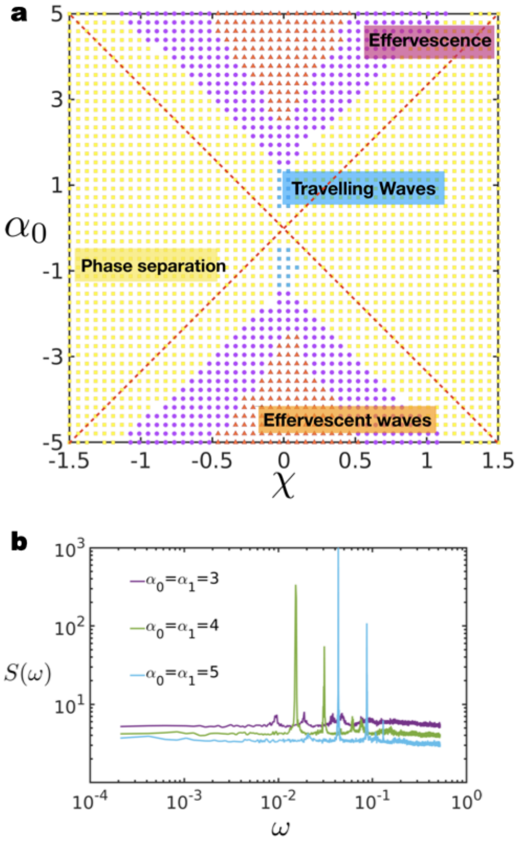

FIG. S6. **State diagram for nonlinear non-reciprocal Cahn-Hilliard dynamics.** **a**, The state diagram for $\alpha_0 = \alpha_1$ and the coefficient for reciprocal interaction χ is obtained by running simulations in two dimensions varying χ and α_0. We observe the steady-states that are summarised in Fig. S5. The steady-state is thus no longer controlled by the exceptional points alone but by the competition between the two possible dynamical states - the travelling waves and the droplets. **b**, Power spectrum for nonlinear non-reciprocal Cahn-Hilliard dynamics. We choose $\alpha_0 = \alpha_1$ and $\langle \phi_i \rangle = 0$ to calculate the power spectrum in the steady-state. We effervescence with (for $\alpha_0 = 3$) and without a wave ($\alpha_0 = 4, 5$).

and

$$\alpha = \alpha_0 - \alpha_1(\phi_1^2 + \phi_2^2); \tag{S8}$$

The topology of the sate diagram reported in [1] is preserved but the transition between the phase separated and the oscillatory states are altered. Notice that the dynamical matrix for the linearized dynamics for the homogeneous reference state has imaginary eigenvalues in the region of the parameter space bound by the red lines.

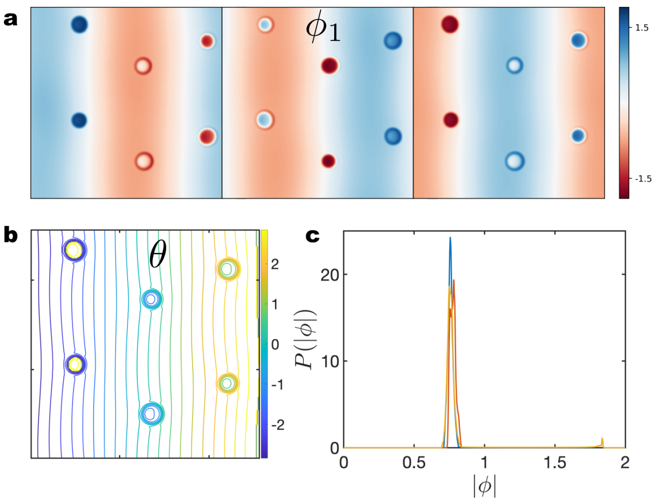

FIG. S7. **Droplets and waves.** **a**, Snapshots of the field ϕ_1 at three different times showing rounded droplets submerged in a background of a ravelling wave for $\langle \phi \rangle = 0 + i0$, $\alpha_0 = 7$, and $\alpha_1 = 2$. **b**, The phase field θ corresponding to the Fig. S7a shows nearly straight lines corresponding to the waves and the circular profile within a droplet. **c**, The probability distribution of the modulus showing two sharp peaks - the one at $|\phi| < 1$ for the wave and the second at $|\phi| = \sqrt{7/2}$ for the droplet. In the spatiotemporally chaotic states, the peaks broaden corresponding to unsteady waves and fluctuating droplets, as seen in Fig. 3b in the main text. We also note that in the steady-state a single size is selected and the oscillatory motive is lost.

VI. STABLE WAVES AND DROPLETS

We observe stable waves *and* waves when $\alpha_0 > \alpha_1$. A defining feature of effervescence is the distribution of the field $|\phi|$ that for the simplified theory in Eq. (5). $P(|\phi|)$ has a bimodal structure Fig. 3b in the main text whose significance we can understand by considering what it looks like in the non-chaotic phases. For a travelling waves the distribution has a sharp peak at the amplitude of the wave while for steady travelling waves with stable droplets it two peaks, one corresponding to the amplitude of the wave and the other to the value of $|\phi|$ where the strength of non-reciprocity precisely vanishes (see Fig. S7), i.e. the second peak corresponds to the droplets. This suggests that a bimodal structure with broad peaks arises when neither the droplets nor the travelling wave solution coexist but neither is stable.

[1] S. Saha, J. Agudo-Canalejo, and R. Golestanian, Scalar active mixtures: The nonreciprocal cahn-hilliard model, Phys. Rev. X **10**, 041009 (2020).

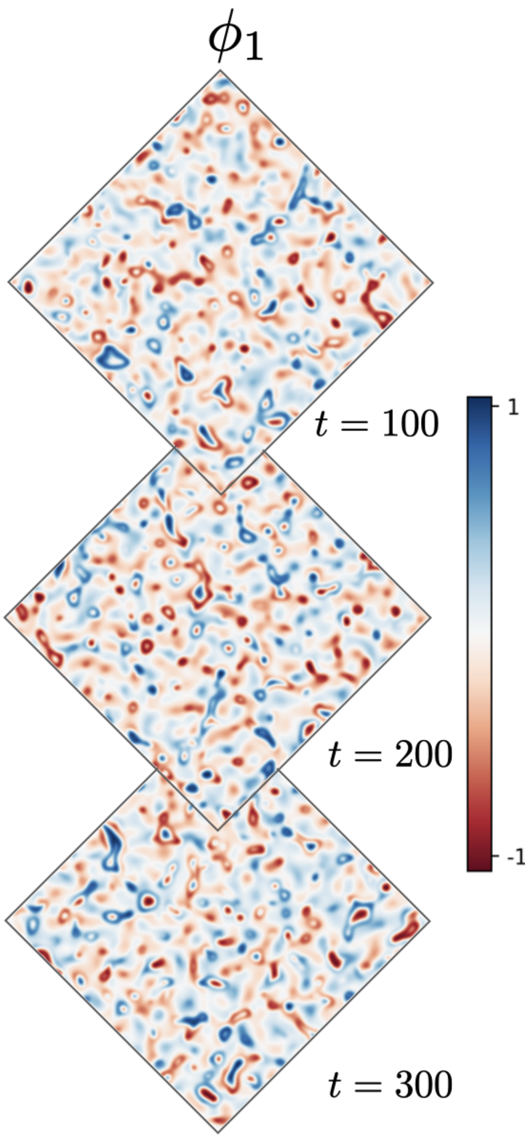

FIG. S8. **Dynamics in the effervescent state.** For $\alpha_0 = 2.3$ and $\alpha_1 = 4.6$ we observe effervescence without an accompanying travelling wave. Here we show snapshots of the field ϕ_1 at times indicated to show that absence of a travelling density wave in contrast to Fig. 1a in the main text.

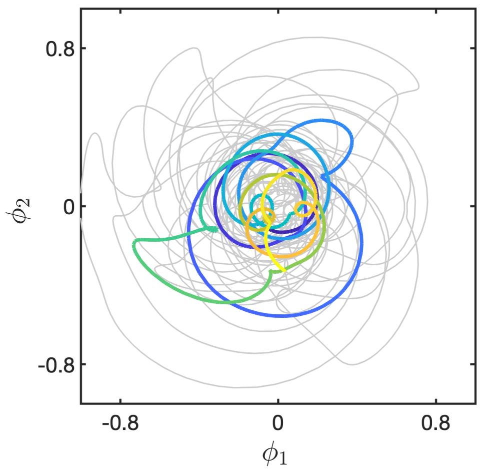

FIG. S9. **Dynamics in the effervescent state.** Here we illustrate the concept of σ with an example. For $\alpha_0 = 2.3$ and $\alpha_1 = 4.6$ we observe effervescence without an accompanying travelling wave, see Fig.1 c-d in the main text. At a fixed point in space the fields $\phi_{1,2}$ trace a 'local' limit cycle. The faint gray line shows the space filling trajectory over $400\Gamma^{-1}K^{-1}$ while the colored track is for a quarter of the full time. The area of the boundary enclosing the local limit cycle calculated via a convex hull constructions and averaged over all points in space is σ. Note that for steady travelling waves, the limit cycle is a circle centred at origin and with radius $\rho_{\boldsymbol{q}}$ while it is precisely a point for a bulk-phase separated state.
