# Supplementary material for: Effervescence in a binary mixture with nonlinear non-reciprocal interactions
Source: Nat Commun. 2025 Aug 7;16:7310. doi: 10.1038/s41467-025-61728-8 (PMC12332134; doi:10.1038/s41467-025-61728-8)
Supplement: Supplementary file 2 — Description of Additional Supplementary Files [file 41467_2025_61728_MOESM2_ESM.pdf]

Description of Additional Supplementary Files

Supplementary Movie 1: Movie showing the evolution of fields phi1 and the modulus |phi| of the complex field phi for alpha0 = 4 and alpha1 = 5. (Video corresponding to Fig. 1a in the main text).

Supplementary Movie 2: Movie showing the evolution of fields phi_1 and the modulus |\phi| of the complex field phi for alpha0 = 2.3 and alpha1 = 4.6. (Video corresponding to Fig. 1b-c in the main text).

Supplementary Movie 3: Movie showing stable round droplets coexisting with a travelling wave for alpha0 = 7 and alpha1 = 2. (Video corresponding to Fig. S7 in the Supplementary Information).

Supplementary Movie 4: Movie showing the evolution of fields phi1 and phi2 of the complex field phi for alpha0 = alpha1 = 4 and phi = 0.25 + i0.25. The average composition can be used to change the dynamical steady state. (Video corresponding to Fig. 8a in the main text).

Supplementary Movie 5: Movie showing the evolution of fields phi1 and phi2 of the complex field phi for alpha0 = alpha1 = 4 and phi = 0.4 + i0. The average composition can be used to change the dynamical steady state. (Video corresponding to Fig. 8b in the main text).
